# Influence of mesenchymal stem cells on metastasis development in mice *in vivo*

**DOI:** 10.1186/s13287-015-0003-7

**Published:** 2015-02-27

**Authors:** Aleksandra V Meleshina, Elena I Cherkasova, Marina V Shirmanova, Natalia V Klementieva, Ekaterina V Kiseleva, Ludmila В Snopova, Natalia N Prodanets, Elena V Zagaynova

**Affiliations:** Nizhny Novgorod State Medical Academy, Minin and Pozharsky Square, 10/1, Nizhny, Novgorod, 603005 Russia; Nizhny Novgorod State University, Gagarin Avenue, 23, Nizhny, Novgorod 603950 Russia; Koltzov Institute of Developmental Biology of Russian Academy of Science, Vavilova st., 26, Moscow, 119334 Russia

## Abstract

**Introduction:**

In recent years, mesenchymal stem cells (MSCs) have been demonstrated to play an important role in carcinogenesis. However, the effect of MSCs on tumor and metastasis development and the mechanisms underlying the interaction of cancer and stem cells are not completely understood. This study investigated the effect of MSCs on breast cancer metastasis formation by using the methods of *in vivo* fluorescence and luminescence imaging.

**Methods:**

MSCs were isolated from bone marrow of normal donors, characterized, and genetically labeled with luciferase (luc2). The effects of MSCs on MDA-MB-231 cancer cell proliferation were evaluated in conditioned medium from MSCs. To generate lung metastases, MDA-MB-231 cells stably expressing red fluorescent protein Turbo FP650 were injected intravenously into nude mice. On day 10 after the cancer cell injection, mice were injected via the tail vein with MSCs-luc2 cells (the MET + MSCs group). Animals that received the injection of MDA-MB-231-Turbo FP650 alone (the MET group) and no injections (the intact control group) served as controls. Fluorescence and bioluminescence imaging was performed for monitoring of the metastasis formation and MSC distribution in the recipient’s body.

**Results:**

We found that the proliferative activity of the cancer cells in the presence of MSC conditioned medium was lower than that of the cells grown in conventional culture medium. The metastasis formation in the MET + MSCs group was delayed in time as compared with the MET group. Macroscopic and histological examination of isolated lungs 8 weeks after cancer cell injection showed that the total number of metastases in animals of the MET + MSCs group was significantly lower. Using bioluminescence imaging *in vivo*, we found that MSCs-luc2 cells survived in the host animal for at least 7 weeks and re-migrated to the lung 6 to 7 weeks after injection. Immunohistochemical analysis revealed the presence of MSCs-luc2 in metastases and lung tissue.

**Conclusions:**

Long-term *in vivo* bioluminescence imaging of intravenously injected MSCs-luc2 cells showed distribution of MSCs to the lungs and abdominal organs within the first 2 to 3 weeks and re-migration to the lungs in weeks 6 to 7. It was found that MSCs reduced the proliferative activity of cancer cells *in vitro* and lung metastasis formation in mice.

## Introduction

In the last few years, mesenchymal stem cells (MSCs) have been demonstrated to play an important role in carcinogenesis. It is known that MSCs of different origin migrate into tumors in a manner similar to the way they migrate into injured tissues [1]. The preferential migration of MSCs into tumors has been widely shown for various tumor xenografts, such as melanoma, ovarian carcinoma, breast carcinoma, and hepatocellular carcinoma [2-4]. The prevalent concept of MSC recruitment into tumors describes their mobilization from systemic niches (bone marrow) and subsequent homing to tumor in response to the release of chemotactic agents from cancer cells. However, the effect of MSCs on tumor and metastasis development and the mechanisms underlying the cancer-stem cells interaction are not completely understood.

Under standard culture conditions, MSCs are non-tumorigenic. However, several reports indicate their capability to influence tumor behavior through modification of the tumor microenvironment [5,6]. It has been established that MSCs are actively involved in tumor angiogenesis, in the creation of a niche to support cancer stem cell survival, and in metastatic processes [7]. Cancer cells within a tumor develop in a symbiotic manner with the surrounding stroma and attract MSCs into the tumor microenvironment. MSCs have been shown to facilitate cancer progression [8] and to affect the morphology and proliferation of cancer cells through cell-to-cell interactions as well as through the secretion of chemotactic cytokines and paracrine factors [9].

The conversion of early-stage tumors into invasive malignancies has been shown to be associated with the activation of the epithelial-mesenchymal transition, defined as changes in cell phenotype from an epithelial to a mesenchymal state. The mesenchymal properties promote a detachment of cancer cells from the primary tumor and facilitate their subsequent migration, allowing metastatic progression [10,11]. Karnoub *et al*. [12] have reported that MSCs enhance breast cancer cell motility, invasion, and metastatic potential *in vivo* through CCL5—chemokine (C-C motif) ligand 5—signaling, which confirms that these paracrine interactions play an important role in the MSC-mediated metastatic spread.

Along with stimulation of carcinogenesis, MSCs can inhibit tumor growth that has been shown on glioma and breast cancer cells in cell culture and mice [13,14]. A suppressing effect of MSCs on the development of breast carcinoma has been demonstrated [14-17]. The precise mechanism underlying the antitumor properties of MSCs has not been fully investigated, but it is presumably related to the downregulation of protein kinase B (Akt), nuclear factor-kappa-B (NF-κB), and wingless int (Wnt) signaling pathways [18,19] and paracrine effects of MSCs, such as Dkk1 and Oncostatin M [20-22].

In recent years, *in vivo* optical imaging has been increasingly used to visualize the distribution of transplanted MSCs in the recipient’s body, track their migration to the tumor site, and monitor subsequent proliferation [23]. Transduction of the stem cells with reporter genes encoding bioluminescent or fluorescent protein provides long-term observation of living cell populations in the animal at the whole-body level [24]. The advantages of optical imaging over other *in vivo* imaging modalities, such as magnetic resonance imaging (MRI) or single-photon emission computed tomography/positron emission tomography (PET), are relative simplicity, low cost and high-throughput of equipment, ease of operation, and short image acquisition time.

The aim of the present work was to study the influence of human MSCs on breast carcinoma metastasis development by using *in vivo* bioluminescence and fluorescence imaging. MDA-MB-231 human breast cancer cells were genetically labeled with red fluorescent protein Turbo FP650. Human MSCs isolated from the bone marrow of healthy donors were transfected with enhanced firefly luciferase gene luc2. Initially, cancer cell proliferative activity in conditioned media from MSCs was tested. Then, MSCs-luc2 cells were injected intravenously into nude mice with previously established MDA-MB-231-Turbo FP650 lung metastases. MSCs-luc2 distribution and migration in animal body and metastasis development were monitored *in vivo*. Metastases in the lungs were confirmed by conventional histopathology, and MSCs-luc2 cells were identified by immunohistochemical analysis.

## Methods

### Cell cultures

All procedures were conducted with approval of the ethics committee of the N.K. Koltzov Institute of Developmental Biology. Patients with no notable pathologic history were chosen for this study. Human MSCs were isolated from the bone marrow of normal donors with informed consent in accordance with the institutional guidelines under the approved protocol. Mononuclear cells were separated by centrifugation over a Histopaque gradient (Sigma-Aldrich, St. Louis, MO, USA), suspended in regular growth medium (Dulbecco’s modified Eagle’s medium, or DMEM) supplemented with 10% fetal bovine serum (FBS) (HyClone, a brand of GE Healthcare, Little Chalfont, UK), 0.58 mg/mL L-glutamine (PanEco, Moscow, Russia), and 40 U/mL gentamicin, and plated on culture flasks. After 3 days, non-adherent cells were removed by washing with phosphate-buffered saline (PBS), and monolayers of adherent cells were cultured until they reached confluence. The cells were then trypsinized (0.25% trypsin with 0.1% EDTA) and subcultured.

The cells were immunophenotypically characterized by flow cytometry on the Cell Lab Quanta SC (Beckman Coulter, Brea, CA, USA) for markers common to human MSCs (CD34, CD45, HLA-DR, CD105, CD44, CD54, CD73, and CD90). Cell passages 3 and 4 were used for the experiments. When MSCs were 80% to 90% confluent in the culture flasks, the cells were incubated for 3 to 4 days, and the medium was collected, filtered through a 0.22-μm filter, and stored at −80°C until used as the MSC conditioned medium. The MDA-MB-231 human breast adenocarcinoma cell line stably expressing red fluorescent protein Turbo FP650 (MDA-MB-231-Turbo FP650) was maintained in DMEM supplemented with 10% FBS, 0.32 mg/mL glutamine, and 40 U/mL gentamicin. The cells were incubated at 37°C in 5% CO_2_ at saturated humidity.

### Lentiviral vector generation

pLuc2-N plasmid and lentiviral vector pLVT-1 were used. The fragment corresponding to the luc2 open reading frame was amplified from pLuc2-N matrix and treated by restriction endonuclease Not I with DNA-polymerase I *Escherichia coli* (Klenow fragment). Then, the luc2 fragment was processed by restriction endonuclease Nhe I. pLVT-1 was also initially processed by restriction endonuclease Sal I with DNA-polymerase I *E. coli* (Klenow fragment) and after that by restrictase Nhe I. pLVT-1/Nhe I-Sal I* and fragment luc2/Nhe I-Not I* were ligated and *E. сoli* XL1-Blue cells were transfected by the ligation product in accordance with a standard protocol. Recombinant clones were screened by polymerase chain reaction (PCR) from bacterial colonies by using luc2 gene-specific PCR primers. The results were verified by sequencing.

### Transfection and stable MSCs-luc2 cell line generation

Lentiviral transduction protocol was performed. HEK 293 T cell line was co-transfected by the mixture of lentiviral plasmids, including pLVT-luc2, with the calcium-phosphate technique. Target MSCs were transfected by the lentiviral particles.

### Treatment of MDA-MB-231 cells with conditioned medium from mesenchymal stem cells

For the observation of the effect of conditioned medium from mesenchymal stem cells (CM-MSCs) on cancer cell proliferation, MDA-MB-231 cells were plated at a density of 10 × 10^4^ cells per well on 96-well plates in the mixture of cancer cell culture medium (supplemented with 10% FBS) and CM-MSCs (1:1). Cancer cells grown in the mixture of cancer cell culture medium supplemented with 10% FBS and MSC growth medium without FBS (1:1) served as the control. After culturing for 48 hours, the viability of cells was assessed in 10 wells by 3-(4,-dimethylthiazol-2-yl)-2,5-diphenyl-tetrazolium bromide (MTT) assay. The absorbance values for positively stained cells were measured by using a microplate reader Synergy MX (BioTek, Winooski, VT, USA) at a 570 nm wavelength to determine the optical density.

### Tumor models and mice

All *in vivo* experiments were approved by the ethics committee of the Nizhny Novgorod State Medical Academy. Female athymic *nu/nu* mice, 4 weeks old, weighing 18 to 20 g were used. To generate lung metastases, MDA-MB-231-Turbo FP650 cells (1 × 10^6^) suspended in 100 μL of PBS were injected intravenously into mice through the tail vein [25].

### Fluorescence and bioluminescence imaging

*In vitro*, *in vivo*, and *ex vivo* imaging was carried out by using an IVIS-Spectrum system (Caliper Life Sciences, Hopkinton, MA, USA). To acquire fluorescence images *in vivo*, an excitation wavelength of 605 nm (bandwidth of 30 nm) was used, and the emission of Turbo FP650 was registered at 660 nm (bandwidth of 20 nm). For *in vivo* imaging, animals were anesthetized with 2% isoflurane.

For luciferase assay in MSCs-luc2 cells *in vitro*, the cells were plated on 96-well plates at the initial concentration of 20,000 cells per well with dilution series of 1:2. D-luciferin (150 μg/mL) dissolved in deionized water was added to the wells. The measurements of bioluminescence intensity were taken in 20 minutes when the signal reached a plateau.

For bioluminescence imaging *in vivo*, animals were given an intraperitoneal injection of 150 mg/kg D-luciferin in PBS 40 minutes before imaging. *Ex vivo* fluorescence and bioluminescence imaging of all organs was performed immediately after the sacrifice of animals. Fluorescence and bioluminescence signals were quantified by using Living Image 4.2 software (Caliper Life Sciences, Hopkinton, MA, USA).

### Effect of mesenchymal stem cells on metastasis development *in vivo*

On the 10th day after the i.v. injection of the MDA-MB-231-Turbo FP650 cells mice were injected via the tail vein with 1 x 10^6^ MSCs-luc2 cells (group “MET + MSCs”). Animals with injection of the MDA-MB-231-Turbo FP650 cells alone (group “MET”) and animals without any injections (group “intact control”) served as controls. Each group included three to five mice.

Fluorescence and bioluminescence imaging was performed *in vivo* prior to injection of cancer cells and MSCs 5 and 24 hours after injection of MSCs and then once a week for 8 weeks. Eight weeks after cancer cell injection, the animals were sacrificed by cervical dislocation, and fluorescence and bioluminescence imaging of tissues *ex vivo* was carried out for identification metastases and MSCs correspondingly. The number of lung metastases was counted under a Leica M60 stereo microscope (Leica, Wetzlar, Germany) immediately after excision. The lungs were fixed in 10% neutral-buffered formalin for further histological and immunohistochemical examination.

### Histology and immunohistochemistry

For histological examination, lungs were surgically removed and fixed in 10% neutral-buffered formalin, dehydrated, and embedded in paraffin in accordance with standard protocol. To verify metastases, paraffin sections 5- to 7-μm thick were stained with hematoxylin-eosin. Immunohistochemistry (IHC) study was accomplished on the paraffin sections by the horseradish-peroxidase method with high-temperature citrate (рН 6.0) buffer antigen unmasking. Antiffluc IHC was performed by using 1:500 dilution. For luc2 antigene detection, primary firefly luciferase rabbit anti-photinus pyralis polyclonal antibodies (LifeSpan Biosciences, Inc., Seattle, WA, USA) were used. To detect primary antibody, ImmPress Reagent Anti-Rabbit Ig Peroxidase (Vector Laboratories, Burlingame, CA, USA) was used. Vector NovaRed substrate (Vector Laboratories) was used as a substrate for peroxidase. Furthermore, the sections were stained with hematoxylin. Negative controls were also tested in IHC reactions to exclude non-specific binding of primary and secondary antibodies. IHC stained sections were examined at 20- and 40-fold magnifications by using an Olympus X71 microscope (Olympus, Tokyo, Japan).

### Statistical analysis

The results are presented as mean and standard deviation. The Mann-Whitney *U* test was used to establish the significance of differences between groups. Differences were considered statistically significant when the *P* value was less than 0.05.

## Results

### Characterization of MSCs-luc2

**Figure 1:**
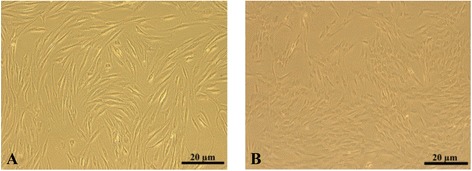
**Мicroscopic images of mesenchymal stem cells (MSCs) in transmitted light. (А)** Parental MSCs. **(B)** MSCs-luc2 cells. luc2, luciferase.

It was shown by microscopic examination and flow сytometry that isolated MSCs had the phenotype of MSCs (Figure 1A and Table 1). MSCs were uniformly positive for CD105, CD90, СD73, CD44, and CD54 and negative for CD34, CD45, and HLA-DR.

**Table 1:** **Fluorescence-activated cell sorting analysis of mesenchymal stem cells**

**Cell marker**	**Marker availability, percentage**
CD34 (sialomucin)	1.290 ± 0.001
CD44 (hyaluronic acid receptor)	90.050 ± 0.006
CD45 (leucocyte common antigen)	0.460 ± 0.001
CD73 (5′-terminal nucleotidase)	93.030 ± 0.040
CD90 (Thy-1)	98,880 ± 0.001
CD54 (intercellular adhesion molecule 1)	47.830 ± 0.014
CD105 (endoglin)	97.130 ± 0.002
HLA-DR (major histocompability complex II)	1.600 ± 0.001

The data are expressed as mean ± standard deviation. n = 3.

Molecular cloning resulted in lentiviral vector containing optimized firefly luciferase luc2 gene. As a result of lentiviral transfection, the MSCs-luc2 stem cell line stably expressing bioluminescent marker was created. After stable transfection with the luc2 gene, MSCs-luc2 cells retained normal morphology (Figure 1B).

**Figure 2:**
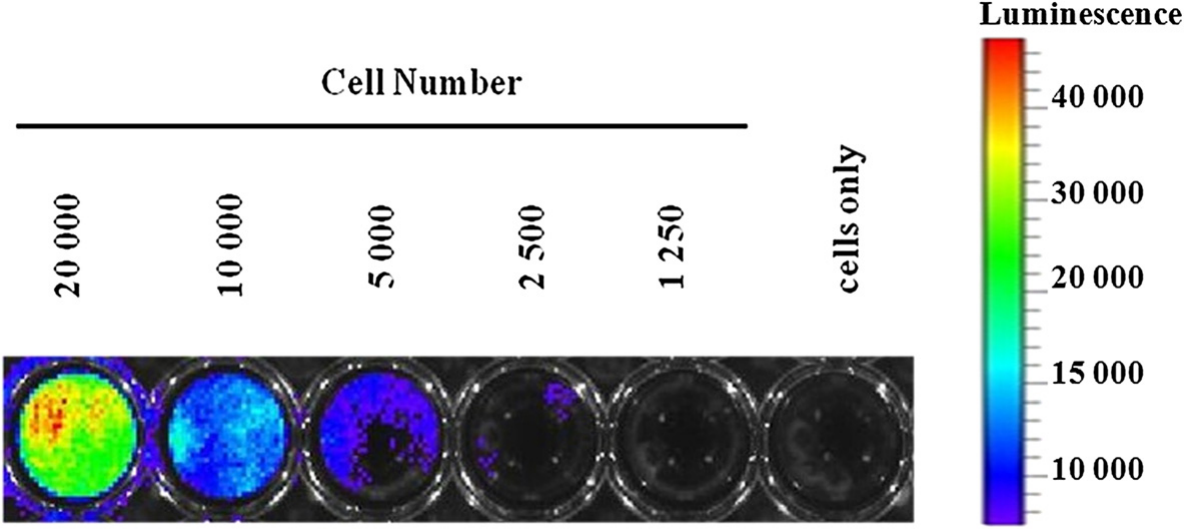
**Bioluminescence of MSCs-luc2.** The highest signal corresponds to the concentration of 20,000 cells per well. Next, wells are 1:2 serial dissolutions. Last, well contains cells only without D-luciferin. luc2, luciferase; MSC, mesenchymal stem cell.

For *in vitro* assessment of bioluminescent signal intensity, a series of diluted MSCs-luc2 cells was prepared in culture plate (Figure 2). Quantitative assessment showed that cells responded to substrate adding adequately. The MSCs-luc2 cells were cultured in several successive passages (over 10), preserving bioluminescent properties.

### MDA-MB-231 cell growth in conditioned media

**Figure 3:**
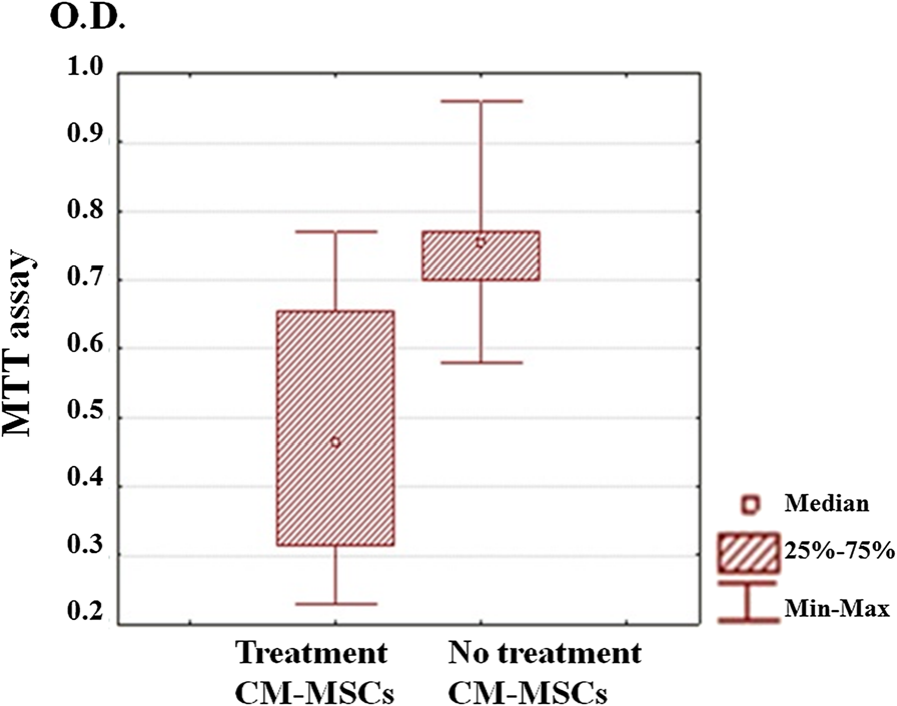
**MTT assay of MDA-MB-231-Turbo FP650 cells treated and untreated with СM-MSCs.** CM-MSC, conditioned medium from mesenchymal stem cells; MTT, 3-(4,-dimethylthiazol-2-yl)-2,5-diphenyl-tetrazolium bromide; O.D., optical density.

To study the influence of MSCs on breast adenocarcinoma MDA-MB-231-Turbo FP650 cells *in vitro*, the cancer cells were grown for 48 hours in CM-MSCs. The MTT test showed that CM-MSCs inhibited proliferation of the cells. Proliferative activity of the MDA-MB-231-Turbo FP650 cells in the presence of CM-MSCs was lower than that of the cells grown in conventional culture medium (*P* <0.05). These data suggest that soluble factors produced by MSCs are responsible for the inhibition of cancer cell growth (Figure 3).

### Influence of mesenchymal stem cells on lung metastasis development in mice

Injection (i.v.) of MDA-MB-231 cells into immunodeficient mice is known to result in metastasis formation in lungs [25]. MDA-MB-231-Turbo FP650 metastasis development in mice that received or did not receive MSCs was monitored during 8 weeks by fluorescence imaging *in vivo*. Fluorescence imaging *ex vivo* and histological analysis of the lung tissue were performed thereafter.

**Figure 4:**
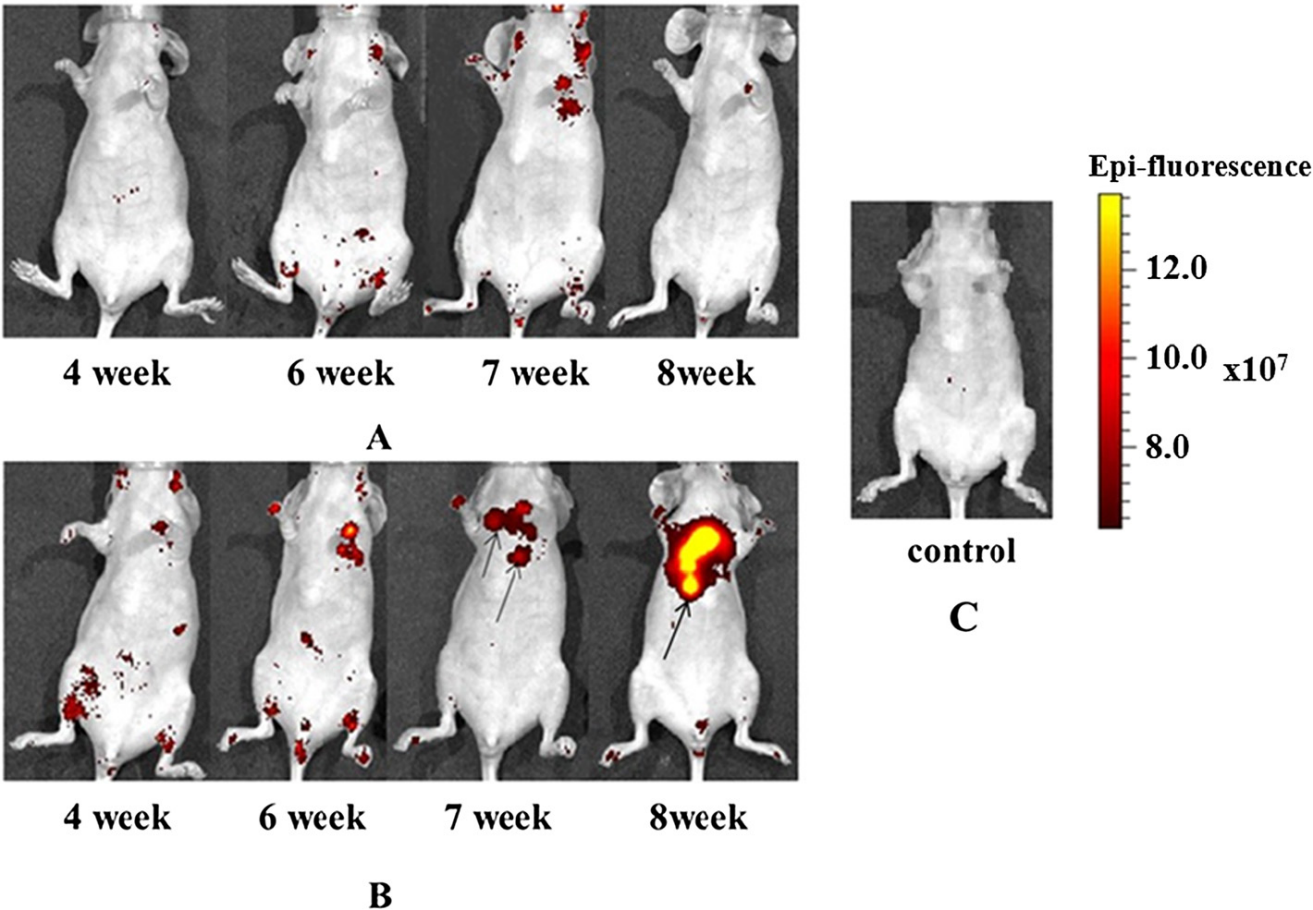
***In vivo*** **imaging of metastasis development in animals. (A)** ‘MET + MSCs’ group. **(B)** ‘MET’ group. **(C)** ‘Intact control’ group. Metastases are shown by arrows. MET, MSC, mesenchymal stem cell.

**Figure 5:**
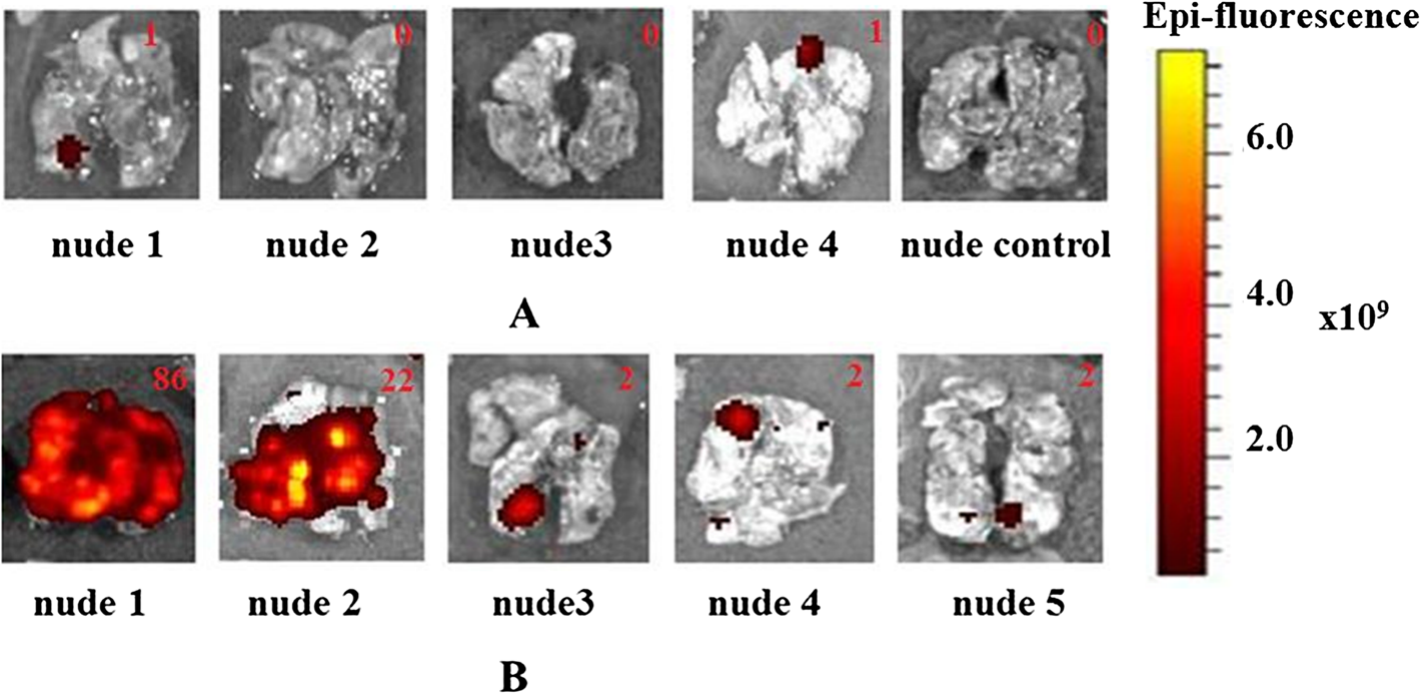
***Ex vivo*** **imaging of metastases in the lungs of animals. (A)** ‘MET + MSCs’ group and ‘intact control’ group. **(B)** ‘MET’ group. Numbers indicate the quantity of metastases detected by macroscopic examination. MET, MSC, mesenchymal stem cell.

In the ‘MET’ group, lung metastases were found in all mice (five out of five). In one animal, fluorescence signal in the chest indicating metastases in the lungs was already recorded 4 weeks after the i.v. injection of cancer cells. Three other mice displayed fluorescence in the lungs *in vivo* by the sixth week (Table 2 and Figure 4A). The presence of metastases in the lungs was confirmed *ex vivo* by fluorescence and macroscopic inspection (Figure 5). In the rest lung fluorescence *in vivo* in one mouse was not registered, however a metastatic locus was found in the lung tissue *ex vivo*. The number of lung metastases in the ‘MET’ group varied from 2 to 86.

**Table 2:** **Lung metastasis development in nude mice**

**Group**	**Time after injection of MDA-MB-231-Turbo FP650 cells, weeks**					
	**3**	**4**	**5**	**6**	**7**	**8**
‘MET + MSCs’	0/4	0/4	0/4	0/4	1/4	1/4
‘MET’	0/5	1/5	1/5	4/5	4/5	4/5

The results are expressed as the number of animals with metastases per total number of animals given injections of cancer cells. MET, ; MSC, mesenchymal stem cell.

In the ‘MET + MSCs’ group, lung metastases were found in two of four animals. In one mouse, *in vivo* fluorescence was first detected 7 weeks after the i.v. injection of cancer cells and increased by the eighth week. Three other mice did not show any fluorescence *in vivo* (Table 2), but *ex vivo* investigation revealed metastasis in one of these three animals (Figures 4B and 5). In two animals, no metastasis was detected by any of the methods; however, histological examination showed odd groups of cancer cells (possibly too small to be seen by fluorescence imaging) in the lungs of one of them. It is important that the number of lung metastases in the ‘MET + MSCs’ group was significantly fewer than in the ‘MET’ group.

### Histopathology

**Figure 6:**
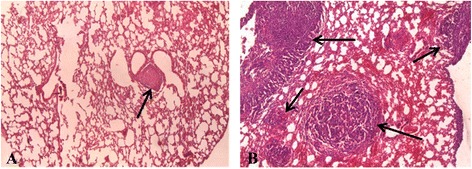
**Histopathological analysis of lung tissue.** Breast cancer metastases are shown by arrows. The image size is 235 × 175 μm. **(A)** ‘MET + MSCs’ group. **(В)** ‘MET’ group. Staining: hematoxylin-eosin. MET, MSC, mesenchymal stem cell.

Through histological analysis of lung tissue, metastases were found in the areas of intense fluorescence (Figure 6). The metastases were represented by small foci consisting of polymorphic cells of various sizes with large nuclei containing diffusely distributed chromatin and one or two nucleoli. The cytoplasm formed a thin ring around the nuclei and had a weak basophilic reaction. Between the tumor cells, blood vessels of the sinusoidal type were observed. Huge polynuclear cancer cells were detected on the periphery of metastasis nodules. Most of the cells in metastases were found in a state of various degrees of dystrophy: from swelling and vacuolization of the cytoplasm to necrosis and hemorrhages (mainly in the center of the nodule).

### Monitoring of MSCs-luc2 migration in mice *in vivo*

Migration of intravenously injected MSCs-luc2 in nude mice with previously induced lung metastases was monitored *in vivo* by means of bioluminescence imaging. Luminescent signal in animals of the ‘MET + MSCs’ group during the follow-up period indicated migration, accumulation, and possible proliferation of MSCs-luc2 cells in corresponding zones. Since the luciferin-luciferase reaction requires ATP and oxygen, only metabolically active, vital cells can be tracked with this method [26].

**Figure 7:**
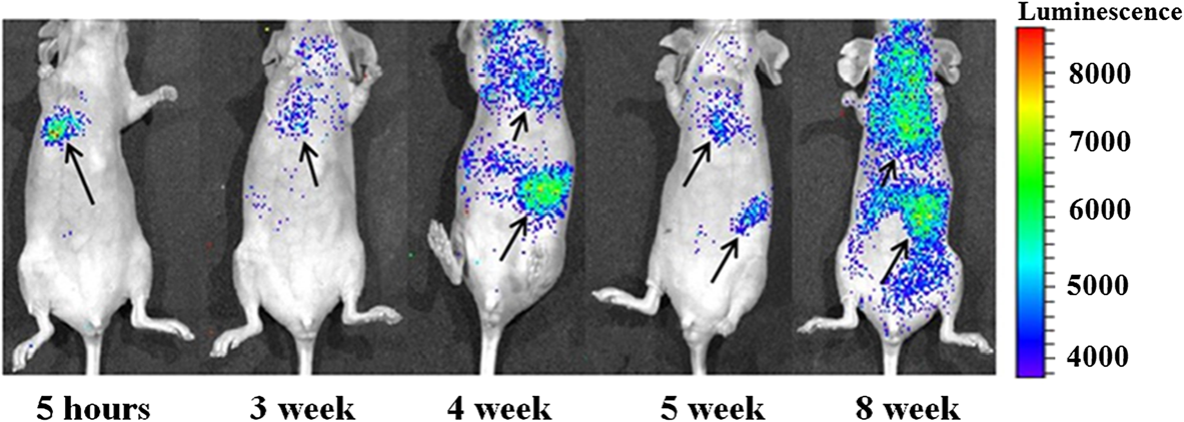
***In vivo*** **bioluminescence imaging of MSCs-luc2 in mice of the ‘MET + MSCs’ group.** MSCs-luc2 cells are shown by arrows. luc2, luciferase; MET, MSC, mesenchymal stem cell.

Intense bioluminescent signal indicating MSCs-luc2 was observed in the chest of all animals from 5 hours to 2 weeks after injection. From 2 to 3 weeks post-injection, the signal in the chest decreased but the signal in the abdomen increased, most likely indicating redistribution of MSCs-luc2. In the fourth and fifth weeks, we observed low or no bioluminescence in the chest and abdominal cavity. In three out of four animals, the signal in the chest increased again in the sixth or seventh week after MSCs-luc2 injection (Figure 7). Bioluminescence imaging *ex vivo* at the end of the seventh week showed the signal from MSCs-luc2 in the lungs, liver, and kidneys.

Therefore, our study showed that MSCs-luc2 cells survived in the host animal for at least 7 weeks after i.v. injection and re-migrated to the lungs. It is essential that re-migration of MSCs-luc2 to the lungs was typical for mice with single lung metastases.

### Immunohistochemistry

**Figure 8:**
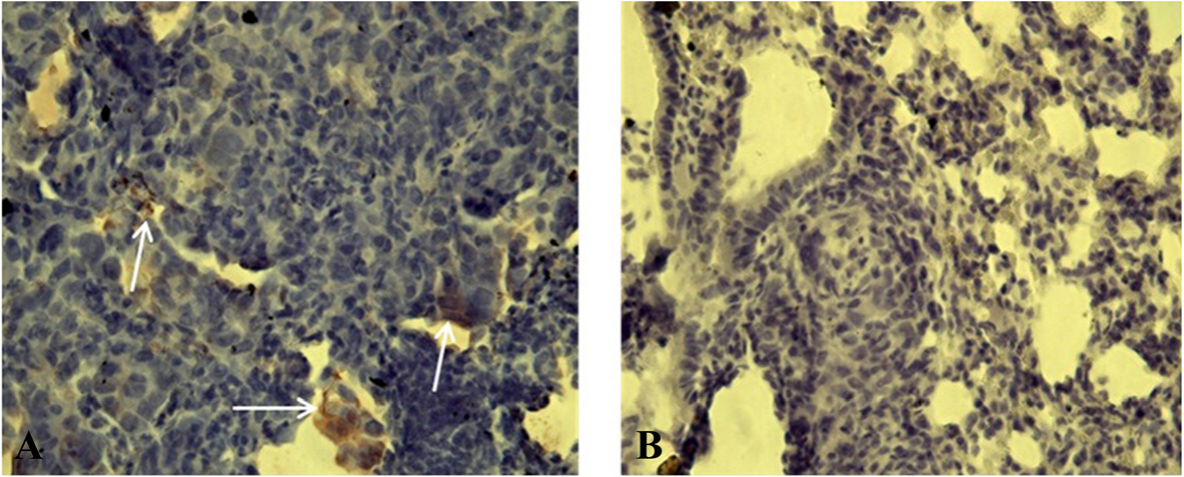
**Immunohistochemistry for ffLuc.** The image size is 316 × 237 μm. **(А)** Mesenchymal stem cells (MSCs) (shown by arrows) in lung metastasis of mouse of the ‘MET + MSC’ group. **(В)** Lung tissue of mouse of the ‘MET’ group. MET,

Immunohistochemical analysis was performed on lung tissue sections of animals of the ‘MET + MSCs’ group to identify MSCs-luc2. Lung tissue of mice of the ‘MET’ group was used as a control. Anti-ffLuc + staining was observed mainly in the metastases, and single MSCs-luc2 cells were found in the lung tissue adjacent to metastatic foci (Figure 8).

## Discussion

In this study, we investigated the effect of intravenously administered human bone marrow MSCs on breast adenocarcinoma metastasis development in nude mice. MSCs were genetically labeled with optimized firefly luciferase luc2 and MDA-MB-231 cancer cells, with red fluorescent protein Turbo FP650 to conduct bioluminescence and fluorescence imaging simultaneously. MDA-MB-231 cells stably expressing Turbo FP650 were injected intravenously into nude mice to induce lung metastases, and MSCs-luc2 cells were administered intravenously 10 days later.

Different works demonstrate the use of dual imaging (fluorescence and bioluminescence) to investigate interaction between stem cells and cancer [27-30]. However, very few works showed the roles of stem cells in breast cancer development by using fluorescence and bioluminescence imaging [31-33]. In those studies, other reporters were used as contrast agents. For example, MSCs labeled with firefly luciferase-enhanced green fluorescence protein (Fluc-eGFP) reporter gene and murine breast cancer cells labeled with renilla luciferase-monomeric red fluorescence protein (Rluc-mRFP) reporter gene were used [31]. Wang and Li [32] studied the interaction between MSCs, labeled with Rluc-RFP-HSV-ttk triple fusion (TF) reporter gene, and breast cancer cells, transduced with Fluc-eGFP double fusion reporter gene. In an article by Leng *et al*. [33], cancer cells were labeled with Fluc and eGFP and MSCs, with a TF gene containing the herpes simplex virus truncated thymidine kinase (HSV-ttk), Rluc, and RFP.

Moreover, in previous studies of breast cancer, dual imaging was used to investigate subcutaneous or fat-pad tumors and spontaneous metastases [32-34]. To the best of our knowledge, the breast cancer metastases induced by i.v. injection of cancer cells were never monitored by using dual imaging.

We showed that MSCs exhibit the potential to suppress the growth of cancer cells *in vitro* and *in vivo*. Cancer cells cultured in CM-MSCs had lower proliferative activity, which testifies to the role of soluble factors in the interaction of MSCs with cancer cells. Intravenously injected MSCs delayed the metastasis formation and decreased the frequency of lung metastases in mice.

The problem of the influence of MSCs on primary tumors and metastases is being actively studied. Along with the works showing the ability of stem cells to stimulate tumor growth [12,35-37], there are the data demonstrating the opposite effect [38-41]. Various effects of MSCs on the development of cancer may account for differences in cancer cell lines used in the works and design of the experiments.

A few works demonstrate the stimulating effect of human bone marrow-derived MSCs on human cancer cells *in vitro* and *in vivo.* Kim *et al*. [42] showed that MSCs increased proliferation of the MCF-7 and MDA-MB-231 cell lines. Fierro *et al*. [43] reported that vascular endothelial growth factor and IL-6 produced by MSCs can stimulate MCF-7 breast cancer cell proliferation *in vitro*. However, in both studies, cocultures of cancer cells and MSCs were used, whereas in our work, cancer cells grew in CM-MSCs.

Karnoub *et al*. [12] showed that MSCs can promote breast tumor metastases in a xenograft model. In that study, MDA-MB-231 cells were mixed with 1.5 × 10^6^ MSCs and injected subcutaneously into immunocompromised mice. Similar results were obtained by Albarenque *et al*. [36] when MDA-MB-231 cells were injected subcutaneously and 1 × 10^5^ MSCs were injected intravenously. In our work, we generated lung metastases by i.v. injection of MDA-MB-231 cells, and MSCs (1 × 10^6^) were also administered intravenously. We suppose that the difference in the results can be explained by different methods of MSC administration, the quantity of MSCs, and the different methods of induction of metastases.

At the same time, our results are consistent with the data about the inhibitory effect of MSCs. The conditioned media of MSCs isolated from human umbilical cord or Wharton’s jelly inhibited proliferation of MDA-MB-231 and other tumor cell lines by arresting the cell cycle in the G_2_/M phase and induced apoptosis *in vitro* [44]. It was also reported that the colony-forming units of MCF-7 in the presence of human dermis tissue CM-MSCs were significantly lower in a dose-dependent manner [14].

Qiao *et al*. [14] showed that tumor formation time was delayed, and the resulting tumor size was significantly smaller in severe combined immunodeficiency (SCID) mice injected with the mixture of MCF-7 and MSCs from human dermis tissues of a dead fetus. It is important that mice that received human umbilical cord MSCs in a mixture with MDA-MB-231 did not have metastases, unlike mice injected with cancer cells alone [14]. Ma *et al*. [15] demonstrated that human umbilical cord MSCs had inhibitory effects on MDA-MB-231 tumor in mice in a dose-dependent manner. A possible mechanism of the inhibition of cancer cell growth is a suppression of the Wnt signaling pathway by MSC secreted factors [21] or promotion of the dormancy of cancer cells [45].

In addition to breast cancer, the inhibitory effect of MSCs was demonstrated on the various types of cancer cell lines (Kaposi’s sarcoma, glioma, lymphoma, and hepatoma) *in vitro* and *in vivo*, which indicates a universality of inhibitory mechanisms of MSCs [46-49].

In our study, we used the model of intravenously induced pulmonary metastases of MDA-MB-231 human breast cancer cells. MDA-MB-231 cells are also known to form spontaneous lung and lymph node metastases from mammary fat-pad tumor. Jenkins *et al*. [50] established multiple spontaneous metastases in the lymph nodes and lungs of nude mice with MDA-MB-231 tumor labeled with firefly luciferase. In our preliminary study on orthotopic MDA-MB-231 tumor xenografts, we injected the nude mice intratumorally with MSCs, and the metastases were not identified either by microscopic examination or by fluorescence imaging of Turbo FP650 (data not shown). This finding potentially indicates the capacity of MSCs for inhibiting not only experimental but also spontaneous metastases and needs further inspection.

Much attention has been paid to the study of MSC migratory capacities *in vivo*. It is known that systemically administered MSCs can migrate to the tumors or wounded tissues [35]. Along with the tissues of interest, injected through the tail vein, human MSCs distribute to the lungs, bone marrow, and lymph nodes first (day 7) and then to the liver and spleen (day 10) [36,51]. Therefore, our data about MSC distribution sites and kinetics are consistent with commonly accepted ideas. However, MSC biodistribution studies *in vivo* are generally limited to 7 to 10 days because the methods of MRI and PET typically used in these studies require labeling of the cells with exogenous contrast agents that are often degraded, diluted, and excreted as cell populations divide [23,24]. In our research, we applied bioluminescence imaging to track MSCs-luc2 cells in living mice and showed survival (and possibly proliferation) of MSCs in the body for at least 7 weeks. We believe this is the first time that such a long-term *in vivo* study on bioluminescent MSCs was performed.

We demonstrated for the first time migration of MSCs-luc2 toward lungs 6 to 7 weeks after i.v. injection, which is presumably associated with attraction of MSCs by metastatic tumor nodules. It is important that bioluminescence in the lungs and IHC for luc2 correlated with the presence of lung metastases. The only mouse in the ‘MET + MSCs’ group that did not show a bioluminescent signal in the lungs at the late period of observation did not have any metastases.

We found that intravenously injected MSCs delayed and inhibited lung metastasis formation in mice. As the MSCs were detected in the tumor tissue, direct interaction between them through soluble factors can be assumed.

## Conclusions

In this work, we studied *in vivo* the effect of human MSCs on the development of metastases of human breast adenocarcinoma MDA-MB-231 cells in mice. Long-term *in vivo* bioluminescence imaging of intravenously injected MSCs genetically labeled with luc2 showed distribution of MSCs to the lungs and abdominal organs within the first 2 to 3 weeks and re-migration to the lungs in 6 to 7 weeks. It was found that MSCs reduced the proliferative activity of cancer cells *in vitro* and lung metastasis formation in mice. Although the exact mechanisms of the inhibiting effect of MSCs on metastasis development need further investigation, our results indicated the attraction of MSCs by cancer cells and interaction between them through soluble factors.

## Abbreviations

CM-MSC: conditioned medium from mesenchymal stem cells
DMEM: Dulbecco’s modified Eagle’s medium
eGFP: enhanced green fluorescent protein
FBS: fetal bovine serum
Fluc: firefly luciferase
HSV-ttk: herpes simplex virus truncated thymidine kinase
IHC: immunohistochemistry
i.v.: intravenous
luc2: luciferase
MET: ᅟ
MRI: magnetic resonance imaging
MSC: mesenchymal stem cell
MTT: 3-(4,-dimethylthiazol-2-yl)-2,5-diphenyl-tetrazolium bromide
PBS: phosphate-buffered saline
PCR: polymerase chain reaction
PET: positron emission tomography
RFP: red fluorescent protein
Rluc: renilla luciferase
TF: triple fusion

## References

[1] Spaeth EL, Klopp A, Dembinski J, Andreeff M, Marini F (2008). Inflammation and tumor microenvironments: defining the migratory itinerary of mesenchymal stem cells. Gene Ther..

[2] Komarova S, Kawakami Y, Stoff-Khalili MA (2006). Mesenchymal progenitor cells as cellular vehicles for delivery of oncolytic adenoviruses. Mol Cancer Ther..

[3] Ren C, Kumar S, Chanda D (2008). Therapeutic potential of mesenchymal stem cells producing interferon-alpha in a mouse melanoma lung metastasis model. Stem Cells..

[4] Niess H, Bao Q, Conrad C (2011). Selective targeting of genetically engineered mesenchymal stem cells to tumor stroma microenvironments using tissuespecific suicide gene expression suppresses growth of hepatocellular carcinoma. Ann Surg..

[5] Stagg J (2008). Mesenchymal stem cells in cancer. Stem Cell Rev..

[6] Roorda BD, TerElst A, Kamps WA, Bont ES (2009). Bone marrow-derived cells and tumor growth:contribution of bone marrow-derived cells to tumor micro-environments with special focus on mesenchymal stem cells. Crit Rev Oncol Hematol..

[7] Bhowmick NA, Neilson EG, Moses HL (2004). Stromal fibroblasts in cancer initiation and progression. Nature..

[8] Hu M, Polyak K (2008). Molecular characterisation of the tumour microenvironment in breast cancer. Eur J Cancer..

[9] Martin FT, Dwyer RM, Kelly J, Khan S, Murphy JM, Curran C (2010). Potential role of mesenchymal stem cells (MSCs) in the breast tumour microenvironment: stimulation of epithelial to mesenchymal transition (EMT). Breast Cancer Res Treat..

[10] Hugo H, Ackland ML, Blick T (2007). Epithelial-mesenchymal and mesenchymal - epithelial transitions in carcinoma progression. J Cell Physiol..

[11] Yang J, Weinberg RA (2008). Epithelial-mesenchymal transition: at the crossroads of development and tumor metastasis. Dev Cell..

[12] Karnoub AE, Dash AB, Vo AP, Sullivan A, Brooks MW, Bell GW (2007). Mesenchymal stem cells within tumor stroma promote breast cancer metastasis. Nature..

[13] Nakamura K, Ito Y, Kawano Y, Kurozumi K, Kobune M, Tsuda H (2004). Antitumor effect of genetically engineered mesenchymal stem cells in a rat glioma model. Gene Ther..

[14] Qiao L, Xu Z, Zhao T, Ye L, Zhang X (2008). Dkk-1 secreted by mesenchymal stem cells inhibits growth of breast cancer cells via depression of Wnt signaling. Cancer Lett..

[15] Ma Y, Hao X, Zhang S, Zhang J (2012). The *in vitro* and *in vivo* effects of human umbilical cord mesenchymal stem cells on the growth of breast cancer cells. Breast Cancer Res Treat..

[16] Stoff-Khalili MA, Rivera AA, Mathis JM, Banerjee NS, Moon AS, Hess A (2007). Mesenchymal stem cells as a vehicle for targeted delivery of CRAds to lung metastases of breast carcinoma. Breast Cancer Res Treat..

[17] Ling X, Marini F, Konopleva M, Schober W, Shi Y, Burks J (2010). Mesenchymal stem cells overexpressing IFN-β inhibit breast cancer growth and metastases through Stat3 signaling in a syngeneic tumor model. Cancer Microenviron..

[18] Qiao L, Zhao TJ, Wang FZ, Shan CL, Ye LH, Zhang XD (2008). NF-kappaB downregulation may be involved the depression of tumor cell proliferation mediated by human mesenchymal stem cells. Acta Pharmacol Sin..

[19] Loebinger MR, Janes SM (2010). Stem cells as vectors for antitumour therapy. Thorax..

[20] Wang ML, Pan CM, Chiou SH, Chen WH, Chang HY, Lee OK (2012). Oncostatin m modulates the mesenchymal–epithelial transition of lung adenocarcinoma cells by a mesenchymal stem cell-mediated paracrine effect. Tumor Stem Cell Biol..

[21] Liu J, Han G, Liu H, Qin C (2013). Suppression of cholangiocarcinoma cell growth by human umbilical cord mesenchymal stem cells: a possible role of Wnt and Akt signaling. PLoS One..

[22] Hou L, Wang X, Zhou Y, Ma H, Wang Z, He J (2014). Inhibitory effect and mechanism of mesenchymal stem cells on liver cancer cells. Tumour Biol..

[23] Zhao C, Tian M, Zhang H (2010). In vivo stem cell imaging. The Open Nucl Med J..

[24] Gao Y, Cui Y, Chan JKY, Xu C (2013). Stem cell tracking with optically active nanoparticles. Am J Nucl Med Mol Imaging..

[25] Zhou Y, Shao G, Liu S (2012). Monitoring breast tumor lung metastasis by U-SPECT-II/CT with an integrinαvβ3-targeted radiotracer 99m Tc-3P-RGD2. Theranostics..

[26] Greer LF, Szalay AA (2002). Imaging of light emission from the expression of luciferases in living cells and organisms: a review. Luminescence..

[27] Ruan J, Song H, Li C, Bao Ch FH, Wang K, Ni J (2012). DiR-labeled embryonic stem cells for targeted imaging of in vivo gastric cancer cells. Theranostics..

[28] Alieva M, Bagó JR, Aguilar E, Soler-Botija C, Vila OF, Molet J (2012). Glioblastoma therapy with cytotoxic mesenchymal stromal cells optimized by bioluminescence imaging of tumor and therapeutic cell response. PLoS One..

[29] Shah K. Imaging neural stem cell fate in mouse model of glioma. Curr Protoc Stem Cell Biol. 2009 Mar;Chapter 5:Unit 5A.1. doi:10.1002/9780470151808.sc05a01s8. 10.1002/9780470151808.sc05a01s8 19306259

[30] Ke C, Liu R, Suetsugu A, Kimura H, Ho JH, Lee OK (2013). In vivo fluorescence imaging reveals the promotion of mammary tumorigenesis by mesenchymal stromal cells. PLoS One..

[31] Wang H, Cao F, De A (2009). Trafficking mesenchymal stem cell engraftment and differentiation in tumor-bearing mice by bioluminescence imaging. Stem Cells..

[32] Wang Y, Li Z (2014). Traceable therapeutic strategy for treatment of breast cancer with mesenchymal stem cells (MSCs). Cancer Cell Microenvironment..

[33] Leng L, Wang Y, He N, Wang D, Zhao Q, Feng G (2014). Molecular imaging for assessment of mesenchymal stem cells mediated breast cancer therapy. Biomaterials..

[34] Mandel K, Yang Y, Schambach A, Glage S, Otte A, Hass R (2013). Mesenchymal stem cells directly interact with breast cancer cells and promote tumor cell growth in vitro and in vivo. Stem Cells Dev..

[35] Cuiffo BG, Karnoub AE (2012). Mesenchymal stem cells in tumor development, Emerging roles and concepts. Cell Adhesion Migration..

[36] Albarenque SM, Zwacka RM, Mohr A (2011). Both human and mouse mesenchymal stem cells promote breast cancer metastasis. Stem Cell Res..

[37] Ljujic B, Milovanovic M, Volarevic V, Murray B, Bugarski D, Przyborski S (2013). Human mesenchymal stem cells creating an immunosuppressive environment and promote breast cancer in mice. Scientific Reports..

[38] Qiao L, Xu Z, Zhao T, Zhao Z, Shi M, Zhao RC (2008). Suppression of tumorigenesis by human mesenchymal stem cells in a hepatoma model. Cell Res..

[39] Li Z, Tan F, Liewehr DJ, Steinberg SM, Thiele CJ (2010). *In vitro* and *in vivo* inhibition of neuroblastoma tumor cell growth by AKT inhibitor perifosine. J Natl Cancer Inst..

[40] Lu YR, Yuan Y, Wang XJ, Wei LL, Chen YN, Cong C (2008). The growth inhibitory effect of mesenchymal stem cells on tumor cells *in vitro* and *in vivo*. Cancer Biol Ther..

[41] Ohlsson LB, Varas L, Kjellman C, Edvardsen K, Lindvall M (2003). Mesenchymal progenitor cell-mediated inhibition of tumor growth in vivo and in vitro in gelatin matrix. Exp Mol Pathol..

[42] Kim J, Escalante LE, Dollar BA, Hanson SE, Hematti P (2013). Сomparison of breast and abdominal adipose tissue mesenchymal stromal/stem cells in support of proliferation of breast cancer cells. Cancer Invest..

[43] Fierro FA, Sierralta WD, Epunan MJ, Minguell JJ (2004). Marrow-derived mesenchymal stem cells: role in epithelial tumor cell determination metastasis. Clin Exp..

[44] Gauthaman K, Yee FC, Cheyyatraivendran S, Biswas A, Choolani M, Bongso A (2012). Human umbilical cord Wharton's jelly stem cell (hWJSC) extracts inhibit cancer cell growth in vitro. J Cell Biochem..

[45] Ono M, Kosaka N, Tominaga N, Yoshioka Y, Takeshita F, Takahashi RU, et al. Exosomes from bone marrow mesenchymal stem cells contain a microRNA that promotes dormancy in metastatic breast cancer cells. Sci Signal. 2014;7:ra63. 10.1126/scisignal.2005231 24985346

[46] Yang C, Lei D, Ouyang W, Ren J, Li H, Hu J (2014). Conditioned media from human adipose tissue-derived mesenchymal stem cells and umbilical cord-derived mesenchymal stem cells efficiently induced the apoptosis and differentiation in human glioma cell lines in vitro. Biomed Res Int..

[47] Abd-Allah SH, Shalaby SM, El-Shal AS, Elkader EA, Hussein S, Emam E (2014). Effect of bone marrow-derived mesenchymal stromal cells on hepatoma. Cytotherapy..

[48] Takahara K, Ii M, Inamoto T, Komura K, Ibuki N, Minami K (2014). Adipose-derived stromal cells inhibit prostate cancer cell proliferation inducing apoptosis. Biochem Biophys Res Commun..

[49] Khakoo AY, Pati S, Anderson SA, Reid W, Elshal MF, Rovira II (2006). Human mesenchymal stem cells exert potent antitumorigenic effects in a model of Kaposi’s sarcoma. J Exp Med..

[50] Jenkins DE, Hornig YS, Oei Y, Dusich J, Purchio T (2005). Bioluminescent human breast cancer cell lines that permit rapid and sensitive in vivo detection of mammary tumors and multiple metastases in immune deficient mice. Breast Cancer Res..

[51] Kidd S, Spaeth E, Dembinski JL (2009). Direct evidence of mesenchymal stem cell tropism for tumor and wounding microenvironments using *in vivo* bioluminescent imaging. Stem Cells..

